# Limb Reconstruction—A New Paradigm in Orthopedic Development

**DOI:** 10.1111/os.14223

**Published:** 2024-09-19

**Authors:** Sihe Qin, Xinlong Ma, Hui Du

**Affiliations:** ^1^ National Research Center for Rehabilitation Technique Aids Affiliated Rehabilitation Hospital Beijing China; ^2^ Tianjin Hospital Tianjin China; ^3^ Beijing Jishuitan Hospital Affiliated to Capital Medical University Beijing China

Limb morphology and functional reconstruction, abbreviated as “limb reconstruction,” has developed globally over the past 40 years based on the Ilizarov tension‐stress law and technical system. It integrates control theory, systemic engineering, and bionic medical perspectives, incorporating modern intelligent and digital technologies, orthotics and prosthetics, external fixators and internal fixation implants. This has expanded the treatment scope to include complex orthopedic trauma repair, deformity correction, limb lengthening, and functional reconstruction of disabilities. Surgical methods have become more minimally invasive, simpler, and more effective while avoiding serious surgical complications.

Many difficult cases, such as limb defects at risk of amputation, systemic multiple joint contracture deformities, and rare orthopedic diseases, which posed significant challenges when treated with classical medical principles and even high‐tech methods, could now be addressed successfully using principles of nature imitation, stress regulation, and bionic reconstruction combined with Ilizarov technique. The treatment areas include all tissue structures and functions related to limb movement below the skull.

Limb reconstruction represents the emergence and frontier exploration of a new interdisciplinary field, resulting from cross‐disciplinary cooperation. Through dynamic and comprehensive external regulation, the treatment process and outcomes are, to some extent, under the control of doctors and patients, making it possible to explore scientific research on the origin of life. This is changing and inspiring certain rules in orthopedics and providing a “key” to understanding related disciplines.

The 6th World Congress of the ASAMI‐BR & ILLRS Societies will be held in Beijing from September 17th to 22nd. The journal “Orthopaedic Surgery” (OS) has published a special issue on “Ilizarov Technique and Limb Reconstruction,” featuring 21 professional articles, including one review article, 19 clinical articles and one case report, contributed by experts from China and United States. These articles covered the hot topics and advanced techniques of many limb reconstruction fields, including wonderful strategies for complex calcaneal^1^, ^2^, ^3^ and finger^4^ reconstruction, innovative nerve distraction,^5^ different types of bone transport for ischemic diseases^6^, ^7^ and tibial defects,^8^, ^9^ comprehensive ways of correction of severe equinocavovarus feet^10^, ^11^, ^12^ and refractory lower limb deformities,^13^, ^14^, ^15^, ^16^, ^17^ application of external fixators and minimally invasive surgery in foot and ankle arthritis,^18^, ^19^ and combination of external fixation and microsurgery for wound cover.^20^, ^21^

The papers published in this issue, offering new concepts and techniques for the development of the limb reconstruction discipline, provided an opportunity for orthopedic colleagues worldwide to understand the recent advancements in external fixation and limb reconstruction in mainland China, and delivered an academic gift for the 6th World Congress of the ASAMI‐BR & ILLRS Societies.
